# The genome sequence of the Atlantic cod, *Gadus morhua *(Linnaeus, 1758)

**DOI:** 10.12688/wellcomeopenres.21122.1

**Published:** 2024-04-12

**Authors:** Sissel Jentoft, Ole K. Tørresen, Ave Tooming-Klunderud, Morten Skage, Spyridon Kollias, Kjetill S. Jakobsen, Wellcome Sanger Institute Tree of Life Management, Samples and Laboratory team, Wellcome Sanger Institute Scientific Operations: Sequencing Operations, Wellcome Sanger Institute Tree of Life Core Informatics team, Tree of Life Core Informatics collective, Darwin Tree of Life Consortium

**Affiliations:** 1 Centre for Ecological and Evolutionary Synthesis, Department of Biosciences, University of Oslo, Oslo, Norway; 2 Norwegian Sequencing Centre, Department of Biosciences, University of Oslo, Oslo, Norway

**Keywords:** Gadus morhua, Atlantic cod, genome sequence, chromosomal, Gadiformes

## Abstract

We present a genome assembly from an individual male *Gadus morhua* (the Atlantic cod; Chordata; Actinopteri; Gadiformes; Gadidae). The genome sequence is 669.9 megabases in span. Most of the assembly is scaffolded into 23 chromosomal pseudomolecules. Gene annotation of this assembly on Ensembl identified 23,515 protein coding genes.

## Species taxonomy

Eukaryota; Metazoa; Eumetazoa; Bilateria; Deuterostomia; Chordata; Craniata; Vertebrata; Gnathostomata; Teleostomi; Euteleostomi; Actinopterygii; Actinopteri; Neopterygii; Teleostei; Osteoglossocephalai; Clupeocephala; Euteleosteomorpha; Neoteleostei; Eurypterygia; Ctenosquamata; Acanthomorphata; Paracanthopterygii; Zeiogadaria; Gadariae; Gadiformes; Gadoidei; Gadidae; *Gadus*; *Gadus morhua* (Linnaeus, 1758) (NCBI:txid8049).

## Background

**Figure 1.**
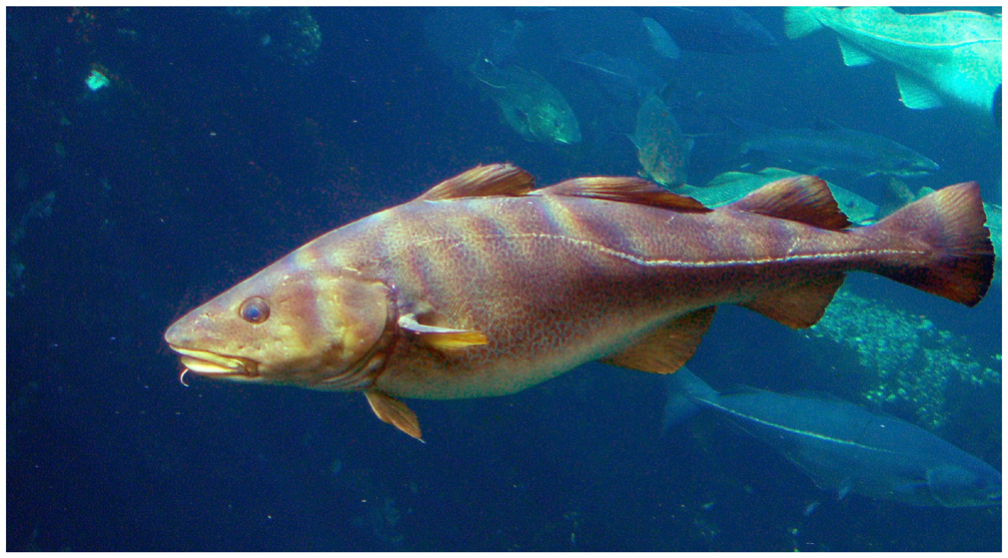
Photograph of *Gadus morhua* (not the specimen used for genome sequencing). Photograph by Hans-Petter Fjeld.

Atlantic cod ( *Gadus morhua*) ( Figure 1) is an ecologically important keystone marine fish species widely distributed throughout the Northern Atlantic Ocean. It has played a major role in fisheries and trade in Northern Europe for hundreds of years ( Star *et al.*, 2017), and is still a highly valued marine resource worldwide. The first version of the Atlantic cod genome – which was released in 2011 – was one of the first vertebrate genomes sequenced using only next generation sequencing technologies. Here, we showed that Atlantic cod has lost major histocompatibility complex (MHC) II genes, which are an essential part of the adaptive immune system ( Star *et al.*, 2011). Additional genome sequencing of a larger selection of Gadiformes and other fish clades further demonstrated that all Gadiformes species lack MHCII and it was caused by a single evolutionary event around 80–100 Mya ( Malmstrøm *et al.*, 2016). Both the original draft genome and the improved second version genome (gadMor2) showed that Atlantic cod has an unusual high proportion of simple tandem repeats (STRs) compared to most other vertebrates ( Star *et al.*, 2011; Tørresen *et al.*, 2017), with putative high evolutionary implications ( Reinar *et al.*, 2023).

Moreover, population genome sequencing in combination with the chromosome anchored reference genome revealed that three larger chromosomal inversions largely distinguish the iconic migratory ecotype the northeast Arctic cod and the stationary non-migratory Norwegian coastal cod ( Berg *et al.*, 2016; Berg *et al.*, 2017). Extending the geographical range further uncovered two genetically distinct co-existing ecotypes in the southernmost fjord systems of Norway: one fjord-type and one more offshore oceanic-type ( Barth *et al.*, 2017; Barth *et al.*, 2019; Jorde *et al.*, 2018), with allele frequency differences in a total of four chromosomal inversions as well as differentiation at the genome-wide level ( Barth *et al.*, 2017; Barth *et al.*, 2019; Sodeland *et al.*, 2016; Sodeland *et al.*, 2022). Recent studies using the gadMor2 genome have shown that the four larger chromosomal inversions most likely have arisen as separate evolutionary events from 400,000 to over a Mya ( Matschiner *et al.*, 2022).

The second version of the genome assembly (gadMor2) was generated using a combination of 454, Illumina and PacBio reads, anchoring scaffolds into chromosomes based on a linkage map ( Tørresen *et al.*, 2017) with fifty-fold larger contig N50 than the first version. However, further improvement in sequencing technologies have enabled even more complete genome assemblies. 

## Genome sequence report

The genome was sequenced from one heterozygous male *Gadus morhua*, individual NEAC_001, a wild-caught male from the Northeast Arctic cod (NEAC) population, and estimated at 8 years of age based on otolith rings. A total of 130-fold coverage in Pacific Biosciences single-molecule long reads, 167-fold coverage in 10X Genomics read clouds, 1416-fold coverage in BioNano reads was generated. Primary assembly contigs were scaffolded with chromosome conformation Hi-C data (76× coverage). Manual assembly curation corrected 429 missing joins or mis-joins and removed 14 haplotypic duplications, reducing the assembly length by 1.74% and the scaffold number by 42.05%, and increasing the scaffold N50 by 23.03%.

**Figure 2.**
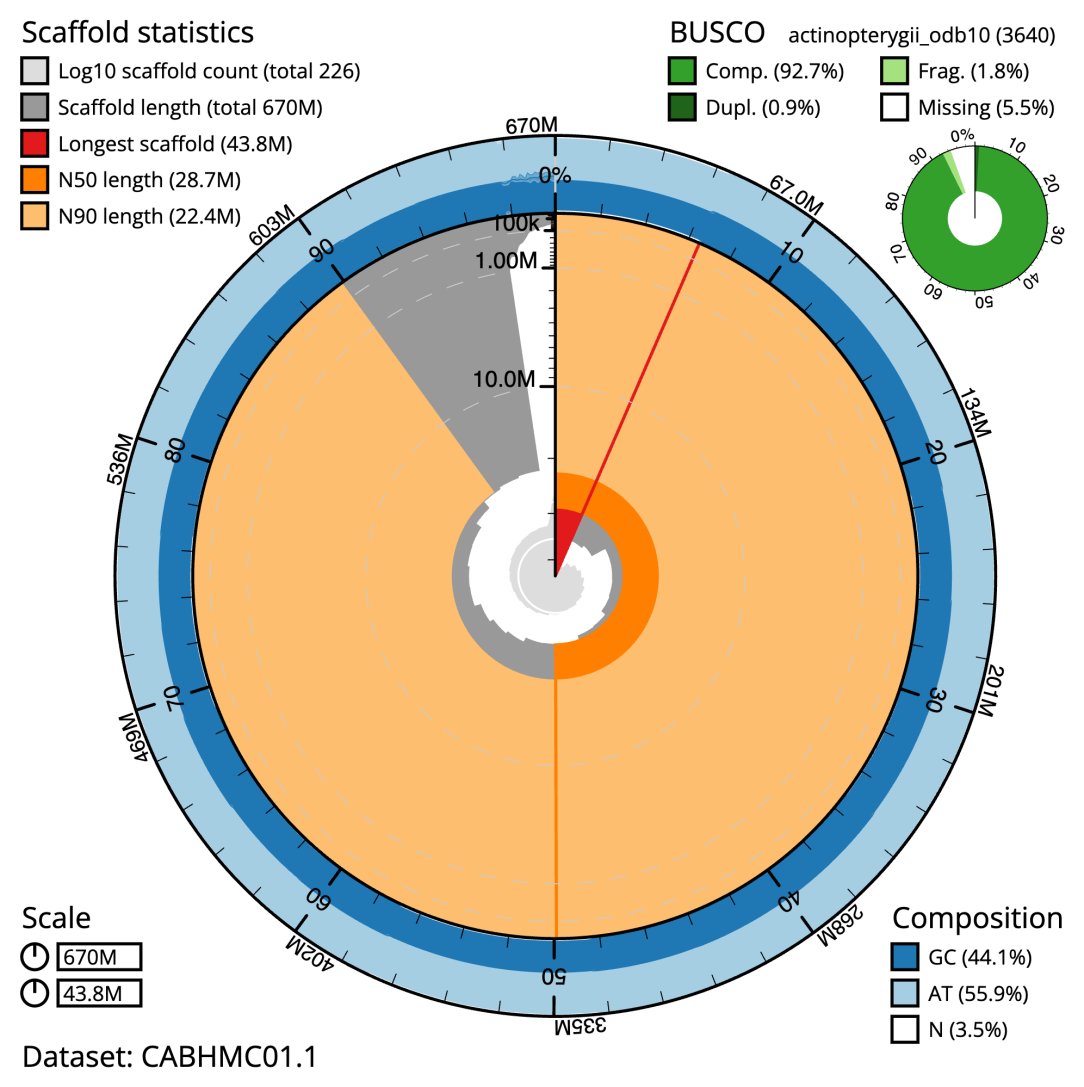
Genome assembly of *Gadus morhua*, gadMor3.0: metrics. The BlobToolKit snail plot shows N50 metrics and BUSCO gene completeness. The main plot is divided into 1,000 size-ordered bins around the circumference with each bin representing 0.1% of the 584,119,146 bp assembly. The distribution of scaffold lengths is shown in dark grey with the plot radius scaled to the longest scaffold present in the assembly (2,763,216 bp, shown in red). Orange and pale-orange arcs show the N50 and N90 scaffold lengths (278,683 and 82,741 bp), respectively. The pale grey spiral shows the cumulative scaffold count on a log scale with white scale lines showing successive orders of magnitude. The blue and pale-blue area around the outside of the plot shows the distribution of GC, AT and N percentages in the same bins as the inner plot. A summary of complete, fragmented, duplicated and missing BUSCO genes in the actinopterygii_odb10 set is shown in the top right. An interactive version of this figure is available at https://blobtoolkit.genomehubs.org/view/Gadus%20morhua/dataset/CABHMC01.1/snail.

**Figure 3.**
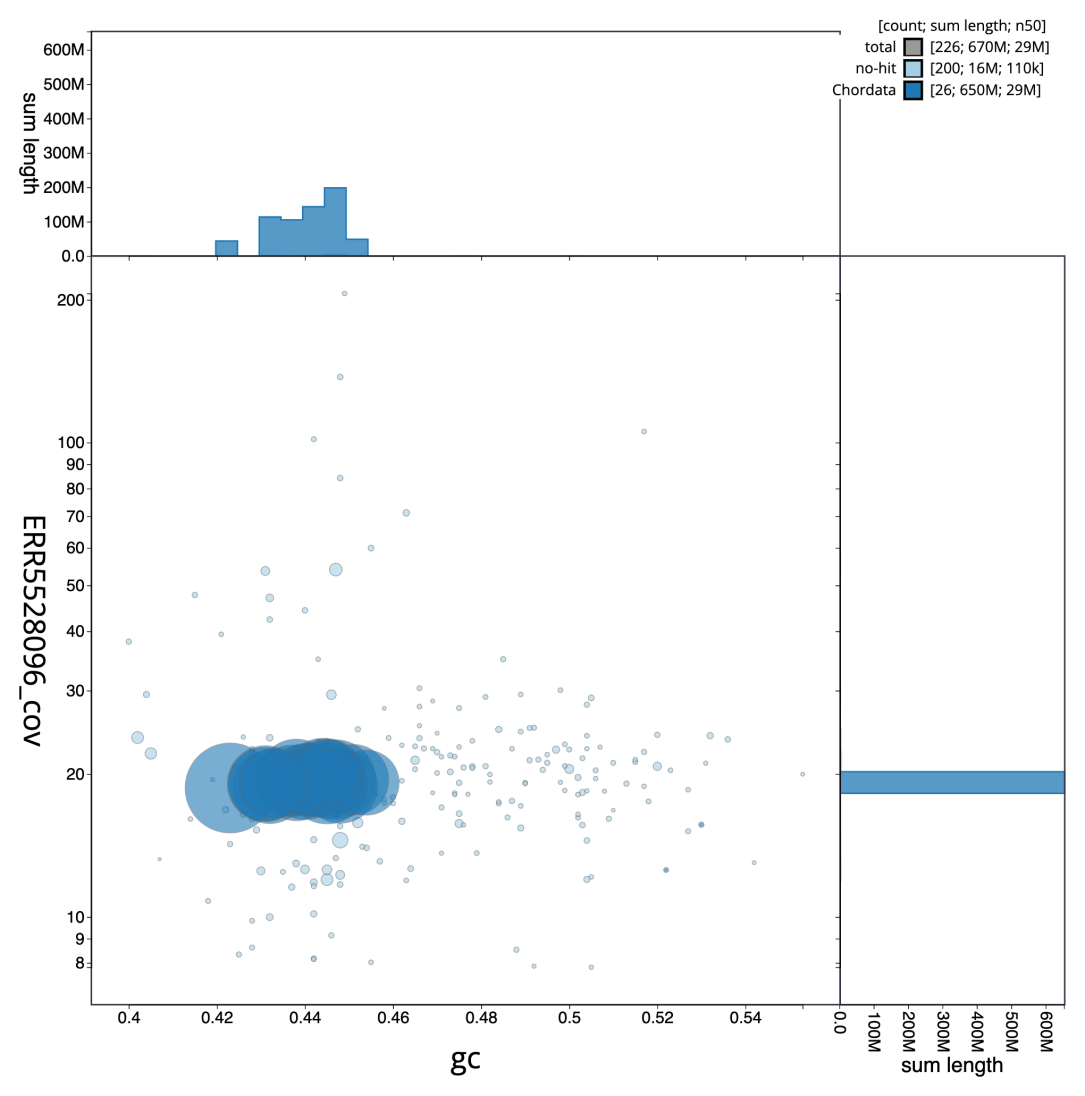
Genome assembly of *Gadus morhua*, gadMor3.0: BlobToolKit GC-coverage plot. Scaffolds are coloured by phylum. Circles are sized in proportion to scaffold length. Histograms show the distribution of scaffold length sum along each axis. An interactive version of this figure is available at https://blobtoolkit.genomehubs.org/view/Gadus%20morhua/dataset/CABHMC01.1/blob.

**Figure 4.**
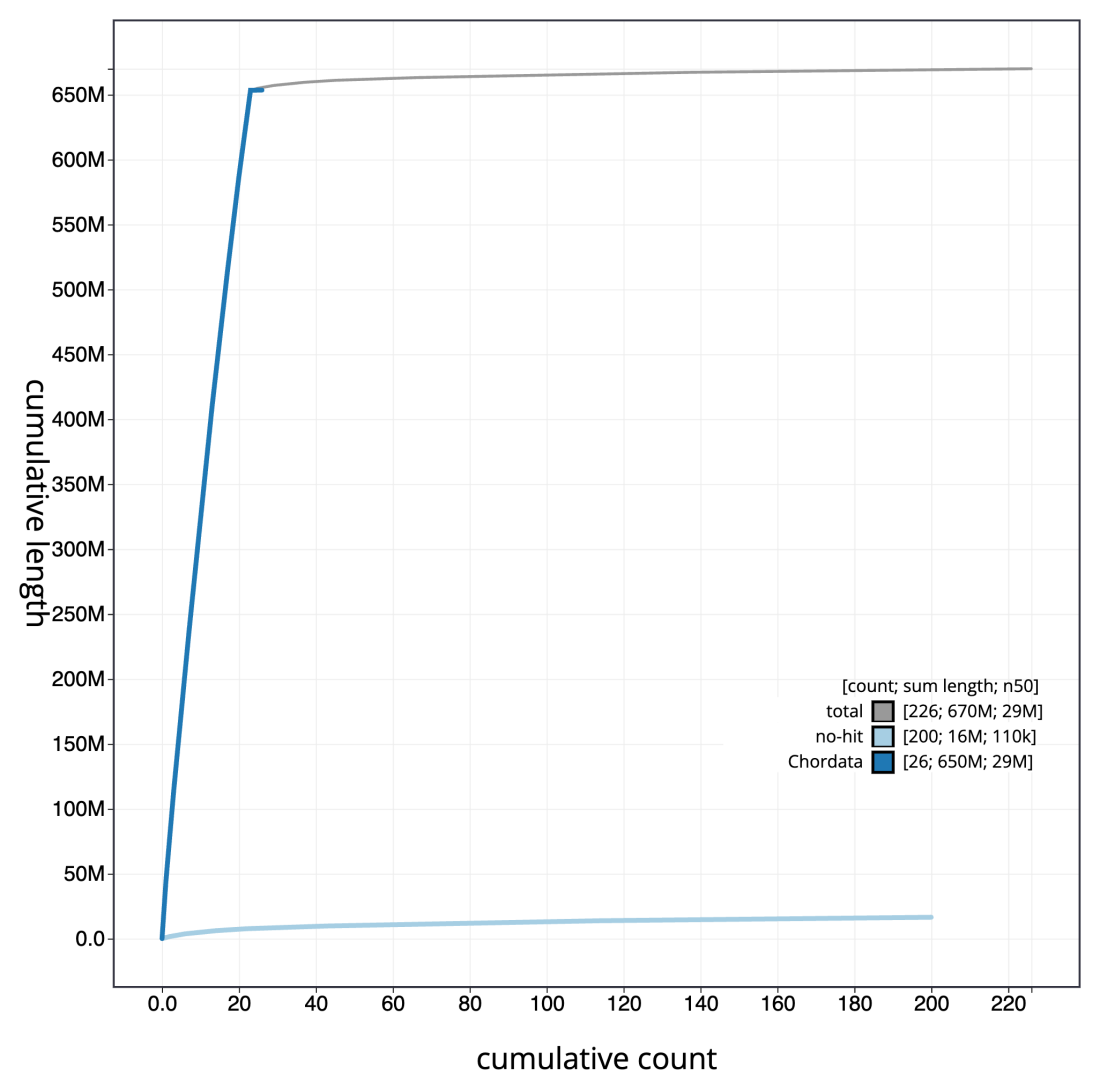
Genome assembly of *Gadus morhua*, gadMor3.0: BlobToolKit cumulative sequence plot. The grey line shows cumulative length for all scaffolds. Coloured lines show cumulative lengths of scaffolds assigned to each phylum using the buscogenes taxrule. An interactive version of this figure is available at https://blobtoolkit.genomehubs.org/view/Gadus%20morhua/dataset/CABHMC01.1/cumulative.

**Figure 5.**
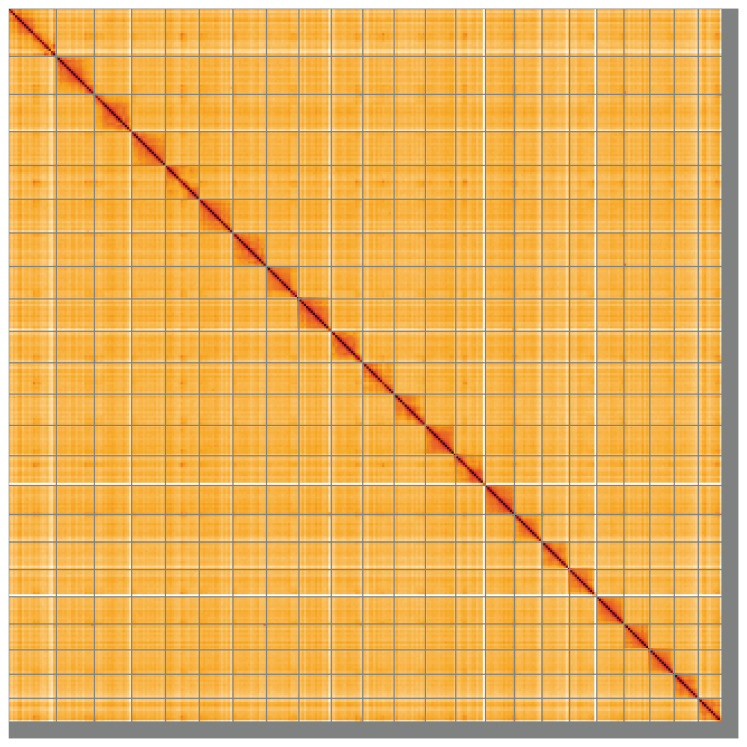
Genome assembly of *Gadus morhua*, gadMor3.0: Hi-C contact map of the gadMor3.0 assembly, visualised using HiGlass. Chromosomes are shown in order of size from left to right and top to bottom. An interactive version of this figure may be viewed at https://genome-note-higlass.tol.sanger.ac.uk/l/?d=N-q-q64SS4-WKerHMnMklw.

The final assembly has a total length of 669.9 Mb in 226 sequence scaffolds with a scaffold N50 of 28.7 Mb ( Table 1). The snail plot in Figure 2 provides a summary of the assembly statistics, while the distribution of assembly scaffolds on GC proportion and coverage is shown in Figure 3. The cumulative assembly plot in Figure 4 shows curves for subsets of scaffolds assigned to different phyla. Most (97.52%) of the assembly sequence was assigned to 23 chromosomal-level scaffolds ( Figure 5). Chromosome-scale scaffolds were named based on a genetic map provided by the Jakobsen lab ( Table 2). While not fully phased, the assembly deposited is of one haplotype. Contigs corresponding to the second haplotype have also been deposited.

**Table 1.** Genome data for *Gadus morhua*, gadMor3.0.

Project accession data		
Assembly identifier	gadMor3.0	
Species	*Gadus morhua*	
Specimen	fGadMor1	
NCBI taxonomy ID	8049	
BioProject	PRJEB33456	
BioSample ID	SAMEA5574046	
Isolate information	fGadMor1	
Assembly metrics *		*Benchmark*
Consensus quality (QV)	38.6	*≥ 50*
*k*-mer completeness	99.56%	*≥ 95%*
BUSCO **	C:92.7%[S:91.8%,D:0.9%], F:1.8%,M:5.5%,n:3,640	*C ≥ 95%*
Percentage of assembly mapped to chromosomes	97.52%	*≥ 95%*
Raw data accessions		
PacBio	ERR7254624–ERR7254628	
10X Genomics Illumina	ERR5528096–ERR5528099	
Genome assembly		
Assembly accession	GCA_902167405.1	
*Accession of alternate haplotype*	GCA_902167395.1	
Span (Mb)	669.9	
Number of contigs	1,441	
Contig N50 length (Mb)	1.0	
Number of scaffolds	226	
Scaffold N50 length (Mb)	28.7	
Longest scaffold (Mb)	30.9	
Genome annotation		
Number of protein-coding genes	23,515	
Number of non-coding genes	5,339	
Number of gene transcripts	68,853	

* Assembly metric benchmarks are adapted from column VGP-2020 of “Table 1: Proposed standards and metrics for defining genome assembly quality” from Rhie *et al.* (2021). ** BUSCO scores based on the actinopterygii_odb10 BUSCO set using v5.3.2. C = complete [S = single copy, D = duplicated], F = fragmented, M = missing, n = number of orthologues in comparison. A full set of BUSCO scores is available at https://blobtoolkit.genomehubs.org/view/Gadus%20morhua/dataset/CABHMC01.1/busco.

**Table 2.** Chromosomal pseudomolecules in the genome assembly of *Gadus morhua*, fGadMor1.

INSDC accession	Chromosome	Length (Mb)	GC%
LR633943.1	1	30.88	45.5
LR633944.1	2	28.73	45.5
LR633945.1	3	30.95	45.5
LR633946.1	4	43.8	45.5
LR633947.1	5	25.3	46.0
LR633948.1	6	27.76	45.5
LR633949.1	7	34.14	45.5
LR633950.1	8	29.71	45.5
LR633951.1	9	26.49	45.5
LR633952.1	10	27.23	45.5
LR633953.1	11	30.71	46.0
LR633954.1	12	30.95	45.5
LR633955.1	13	28.83	45.5
LR633956.1	14	29.59	45.0
LR633957.1	15	28.66	45.5
LR633958.1	16	34.79	45.5
LR633959.1	17	21.72	46.0
LR633960.1	18	24.9	46.0
LR633961.1	19	22.02	45.5
LR633962.1	20	24.84	45.5
LR633963.1	21	22.36	46.0
LR633964.1	22	23.74	46.0
LR633965.1	23	25.24	46.0

The estimated Quality Value (QV) of the final assembly is 38.6 with *k*-mer completeness of 99.56%, and the assembly has a BUSCO v5.3.2 completeness of 92.7% (single = 91.8%, duplicated = 0.9%), using the actinopterygii_odb10 reference set ( *n* = 3,640).

Metadata for specimens, barcode results, spectra estimates, sequencing runs, contaminants and pre-curation assembly statistics are given at https://links.tol.sanger.ac.uk/species/8049.

## Genome annotation report

The *Gadus morhua* genome assembly (GCA_902167405.1) was annotated using the Ensembl rapid annotation pipeline at the European Bioinformatics Institute (EBI). The resulting annotation includes 68,853 transcribed mRNAs from 23,515 protein-coding and 5,339 non-coding genes ( Table 1; https://rapid.ensembl.org/Gadus_morhua_GCA_902167405.1/Info/Index).

## Methods

### Sample acquisition and nucleic acid extraction

The sequenced cod (NEAC_001) was a wild-caught male from the NEAC population, estimated at 8 years of age based on otolith rings. High molecular weight DNA was extracted from i) flash frozen blood (at Sanger) and ii) agarose blood plugs (at UiO) from the NEAC_001. DNA was dissolved overnight in 1 ml of TE-buffer. Quality and quantity of DNA were checked using NanoDrop (NanoDrop Products), PicoGreen Quant-iT™ (Invitrogen) and FLUOstar Optima (BMG Labtech) and through visual inspection of agarose gels.

### Sequencing

PacBio data previously generated on the RSII and Sequel systems by the Jakobsen lab at the University of Oslo, Norway, were combined with data from 5 additional SMRTcells generated at the Wellcome Sanger Institute (WSI). In addition, Chromium 10X Genomics data were generated on the Illumina HiSeqX platform at WSI, and BioNano Saphyr DLE maps were produced for structural variant analysis. Arima Hi-C data were generated from heart and gill tissue at the Jakobsen lab and sequenced on Illumina HiSeq. Raw data can be accessed at GenomeArk.

### Genome assembly, curation and evaluation

The assembly process included the following sequence of steps: initial PacBio assembly generation with Falcon-unzip ( Chin *et al.*, 2016), retained haplotig identification with purge_dups ( Guan *et al.*, 2020), 10X based scaffolding with scaff10x, BioNano hybrid-scaffolding, Hi-C based scaffolding with SALSA2 ( Ghurye *et al.*, 2019), Arrow polishing, and two rounds of FreeBayes ( Garrison & Marth, 2012) polishing. The assembly was checked for contamination and corrected using the gEVAL system ( Chow *et al.*, 2016) as described previously ( Howe *et al.*, 2021). Manual curation was performed using gEVAL, HiGlass ( Kerpedjiev *et al.*, 2018) and Pretext ( Harry, 2022). Chromosome-scale scaffolds were named based on a genetic map provided by the Jakobsen lab.

A Hi-C map for the final assembly was produced using bwa-mem2 ( Vasimuddin *et al.*, 2019) in the Cooler file format ( Abdennur & Mirny, 2020). To assess the assembly metrics, the *k*-mer completeness and QV consensus quality values were calculated in Merqury ( Rhie *et al.*, 2020). This work was done using Nextflow ( Di Tommaso *et al.*, 2017) DSL2 pipelines “sanger-tol/readmapping” ( Surana *et al.*, 2023a) and “sanger-tol/genomenote” ( Surana *et al.*, 2023b). The genome was analysed within the BlobToolKit environment ( Challis *et al.*, 2020) and BUSCO scores ( Manni *et al.*, 2021; Simão *et al.*, 2015) were calculated.

Table 3 contains a list of relevant software tool versions and sources.

**Table 3.** Software tools: versions and sources.

Software tool	Version	Source
BlobToolKit	4.1.7	https://github.com/blobtoolkit/blobtoolkit
BUSCO	5.3.2	https://gitlab.com/ezlab/busco
Falcon-unzip	-	https://github.com/PacificBiosciences/FALCON_unzip
FreeBayes	1.3.1-17-gaa2ace8	https://github.com/freebayes/freebayes
gEVAL	N/A	https://geval.org.uk/
HiGlass	1.11.6	https://github.com/higlass/higlass
Long Ranger ALIGN	2.2.2	https://support.10xgenomics.com/genome-exome/software/pipelines/latest/advanced/other-pipelines
Merqury	MerquryFK	https://github.com/thegenemyers/MERQURY.FK
MitoHiFi	2	https://github.com/marcelauliano/MitoHiFi
PretextView	0.2	https://github.com/wtsi-hpag/PretextView
purge_dups	1.2.3	https://github.com/dfguan/purge_dups
SALSA	2.2	https://github.com/salsa-rs/salsa
sanger-tol/genomenote	v1.0	https://github.com/sanger-tol/genomenote
sanger-tol/readmapping	1.1.0	https://github.com/sanger-tol/readmapping/tree/1.1.0

### Genome annotation

The Ensembl Genebuild annotation system at the EBI ( Aken *et al.*, 2016) was used to generate annotation for the *Gadus morhua* assembly (GCA_902167405.1). Annotation was created primarily through alignment of transcriptomic data to the genome, with gap filling via protein-to-genome alignments of a select set of proteins from UniProt ( UniProt Consortium, 2019).

### Wellcome Sanger Institute – Legal and Governance

The materials that have contributed to this genome note have been supplied by a Darwin Tree of Life Partner. The submission of materials by a Darwin Tree of Life Partner is subject to the **‘Darwin Tree of Life Project Sampling Code of Practice’**, which can be found in full on the Darwin Tree of Life website here. By agreeing with and signing up to the Sampling Code of Practice, the Darwin Tree of Life Partner agrees they will meet the legal and ethical requirements and standards set out within this document in respect of all samples acquired for, and supplied to, the Darwin Tree of Life Project. 

Further, the Wellcome Sanger Institute employs a process whereby due diligence is carried out proportionate to the nature of the materials themselves, and the circumstances under which they have been/are to be collected and provided for use. The purpose of this is to address and mitigate any potential legal and/or ethical implications of receipt and use of the materials as part of the research project, and to ensure that in doing so we align with best practice wherever possible. The overarching areas of consideration are:

•     Ethical review of provenance and sourcing of the material

•     Legality of collection, transfer and use (national and international) 

Each transfer of samples is further undertaken according to a Research Collaboration Agreement or Material Transfer Agreement entered into by the Darwin Tree of Life Partner, Genome Research Limited (operating as the Wellcome Sanger Institute), and in some circumstances other Darwin Tree of Life collaborators.

## Data Availability

European Nucleotide Archive: *Gadus morhua* (Atlantic cod). Accession number PRJEB33456; https://identifiers.org/ena.embl/PRJEB33456 ( Wellcome Sanger Institute, 2021). The genome sequence is released openly for reuse. The *Gadus morhua* genome sequencing initiative is part of the Darwin Tree of Life (DToL) project and the Vertebrate Genomes Project (VGP). The assembly has been deposited in INSDC databases. Raw data and assembly accession identifiers are reported in Table 1.

## Author information

Members of the Wellcome Sanger Institute Tree of Life Management, Samples and Laboratory Team are listed here: https://doi.org/10.5281/zenodo.10066175.

Members of Wellcome Sanger Institute Scientific Operations: Sequencing Operations are listed here: https://doi.org/10.5281/zenodo.10043364.

Members of the Wellcome Sanger Institute Tree of Life Core Informatics team are listed here: https://doi.org/10.5281/zenodo.10066637.

Members of the Tree of Life Core Informatics collective are listed here: https://doi.org/10.5281/zenodo.5013541.

Members of the Darwin Tree of Life Consortium are listed here: https://doi.org/10.5281/zenodo.4783558.
