# Supplementary material for: The genome sequence of the Atlantic cod, Gadus morhua (Linnaeus, 1758)
Source: Wellcome Open Res. 2025 Apr 28;9:189. Originally published 2024 Apr 12. [Version 2] doi: 10.12688/wellcomeopenres.21122.2 (PMC11367075; doi:10.12688/wellcomeopenres.21122.2)
Supplement: Peer review history

# Reviewer report: Reviewer response for version 2

**DOI:** 10.21956/wellcomeopenres.26385.r122748

**Published:** 2025-05-27

**Authors:** Ruiqi Li

**Affiliations:** 1 University of Colorado Boulder, Boulder, Colorado, USA

The authors have addressed all my concerns.

Are sufficient details of methods and materials provided to allow replication by others?

Yes

Is the rationale for creating the dataset(s) clearly described?

Partly

Are the datasets clearly presented in a useable and accessible format?

Yes

Are the protocols appropriate and is the work technically sound?

Yes

Reviewer Expertise:

Genomics

I confirm that I have read this submission and believe that I have an appropriate level of expertise to confirm that it is of an acceptable scientific standard.

# Reviewer report: Reviewer response for version 2

**DOI:** 10.21956/wellcomeopenres.26385.r122747

**Published:** 2025-05-13

**Authors:** Merly Escalona

**Affiliations:** 1 University of California Santa Cruz, Santa Cruz, California, USA

My comments have been addressed. I have no other comments.

Are sufficient details of methods and materials provided to allow replication by others?

Yes

Is the rationale for creating the dataset(s) clearly described?

Partly

Are the datasets clearly presented in a useable and accessible format?

Yes

Are the protocols appropriate and is the work technically sound?

Yes

Reviewer Expertise:

genome assembly

I confirm that I have read this submission and believe that I have an appropriate level of expertise to confirm that it is of an acceptable scientific standard.

# Reviewer report: Reviewer response for version 1

**DOI:** 10.21956/wellcomeopenres.23364.r91124

**Published:** 2024-09-01

**Authors:** Merly Escalona

**Affiliations:** 1 University of California Santa Cruz, Santa Cruz, California, USA

In this study, the authors present a high-quality genome assembly for a male Atlantic cod. The metadata and genome assembly processes provided are comprehensive. Though the metrics do not reach the benchmark values stated in the document, they match those of a high-quality genome.  

 The minor comments presented below agree with the previous reviewer. Please check. 

- The Background reads more like a discussion than an introduction. It introduces other resources, but the reasoning behind a new effort to assemble a genome, while somewhat trivial, is not explained. In addition, it is not clear if some of the results are from previous papers or a result of the current data note.
   
  -  For example, in the Background Section, 4th sentence:
     
    - Here, we showed that Atlantic cod has lost major histocompatibility complex (MHC) II genes, which are an essential part of the adaptive immune system ( Star et al., 2011)
    - Is that statement a result of the current paper? If so, how was this identified? If not, referring to previous studies, maybe the sentence should be rephrased, “ … In Start et al. 2011, it was shown that the Atlantic code has lost…”
- It is not explained whether the mitochondrial genome has been generated, and Table 3 references the MitoHiFi pipeline but does not provide information on the results. If it was generated, please introduce some summary metrics for it.

Are sufficient details of methods and materials provided to allow replication by others?

Yes

Is the rationale for creating the dataset(s) clearly described?

Partly

Are the datasets clearly presented in a useable and accessible format?

Yes

Are the protocols appropriate and is the work technically sound?

Yes

Reviewer Expertise:

genome assembly

I confirm that I have read this submission and believe that I have an appropriate level of expertise to confirm that it is of an acceptable scientific standard, however I have significant reservations, as outlined above.

# Response

**Published:** 2025-03-02

**Authors:** Tree of Life Team Sanger

**Affiliations:** Wellcome Trust Sanger Institute, UK

Thank you for reviewing this data note. We have responded to your points below.

- The Background reads more like a discussion than an introduction. It introduces other resources, but the reasoning behind a new effort to assemble a genome, while somewhat trivial, is not explained. In addition, it is not clear if some of the results are from previous papers or a result of the current data note. For example, in the Background Section, 4th sentence: "Here, we showed that Atlantic cod has lost major histocompatibility complex (MHC) II genes, which are an essential part of the adaptive immune system ( Star et al., 2011)." Is that statement a result of the current paper? If so, how was this identified? If not, referring to previous studies, maybe the sentence should be rephrased, “ … In Start et al. 2011, it was shown that the Atlantic code has lost…”

***Response:** Thank you. We have tried to adjust this to make it clearer that this is referring to the 2011 paper.*

- It is not explained whether the mitochondrial genome has been generated, and Table 3 references the MitoHiFi pipeline but does not provide information on the results. If it was generated, please introduce some summary metrics for it.

***Response:** The mitochondrial genome was not assembled for this project, and we have removed the reference to the MitoHiFi pipeline in the table.*

# Reviewer report: Reviewer response for version 1

**DOI:** 10.21956/wellcomeopenres.23364.r91121

**Published:** 2024-08-31

**Authors:** Leif Andersson

**Affiliations:** 1 Department of Biochemistry and Microbiology, Uppsala University, College Station, Texas, Sweden

This brief report documents the release of a high-quality assembly of the cod genome. This will be a very valuable resource for future work on cod biology. 

**I have only a few comments for minor improvements:**

- Background line 11. The citation to Reinar et al. is a bit misleading since the reader may get the impression that this is a cod paper, but Reinar et al. is a paper on *Arabidopsis *this should be rephrased.
- Your statement that one heterozygous male was sequenced is confusing, all cod are heterozygous at some loci. What do you refer to here? An non-inbred male or a male heterozygous for a particularly important locus?
- Shouldn’t the specimen name (fGadMor1) be the same as the Id number given in the text (NEAC_001)? Please clarify if this is a different individual compared with those used for the previous cod assemblies.
- It would be useful if the authors could comment why the QV score (38.6) does not reach the bench mark (>50) despite the extensive amount of sequence data.
- Table 2. I recommend that a consistent number of decimal places are used for Length in this table.

Are sufficient details of methods and materials provided to allow replication by others?

Yes

Is the rationale for creating the dataset(s) clearly described?

Yes

Are the datasets clearly presented in a useable and accessible format?

Yes

Are the protocols appropriate and is the work technically sound?

Yes

Reviewer Expertise:

Population and Evolutionary Genomics

I confirm that I have read this submission and believe that I have an appropriate level of expertise to confirm that it is of an acceptable scientific standard.

# Response

**Published:** 2025-03-02

**Authors:** Tree of Life Team Sanger

**Affiliations:** Wellcome Trust Sanger Institute, UK

Thank you for reviewing this data note. We have responded to your comments below. 

- Background line 11. The citation to Reinar et al. is a bit misleading since the reader may get the impression that this is a cod paper, but Reinar et al. is a paper on *Arabidopsis *this should be rephrased.

***Response: **Thanks for *notifying us. We are now referring to a study done on teleosts which discusses the role of STRs.

- Your statement that one heterozygous male was sequenced is confusing, all cod are heterozygous at some loci. What do you refer to here? An non-inbred male or a male heterozygous for a particularly important locus?

***Response: **We have removed the reference to a ‘heterozygous male’. It was ment to be coupled to male-heterogametic sex-determination found in cod, but we see that this was misleading.*  

- Shouldn’t the specimen name (fGadMor1) be the same as the Id number given in the text (NEAC_001)? Please clarify if this is a different individual compared with those used for the previous cod assemblies.

***Response:** This particular individual is the same as sequenced and published on in both 2011 and 2017, and it was called NEAC_001 then. When it came into the Sanger/VGP system, it got the specimen name fGadMor1. We have clarified this in the text.*

  

- It would be useful if the authors could comment why the QV score (38.6) does not reach the bench mark (>50) despite the extensive amount of sequence data.

 
***Response:** Many assemblies reach the benchmark of a QV of more than 50 by using ample amounts of PacBio HiFi and comparing to a k-mer database of the same data. When comparing to a k-mer database of Illumina data (for instance Hi-C), this QV score is lower. In this particular project, the cod was sequenced with the older PacBio CLR technology, that is, not the newer HiFi/CCS. This older technology might not reach the same consensus quality as the newer technology and therefore we have this discrepancy. The EBP recommended standard for QV is 40, rather than 50, which we have also corrected here.*

  

- Table 2. I recommend that a consistent number of decimal places are used for Length in this table.

***Response: **We have reduced this to one decimal place.*

# Reviewer report: Reviewer response for version 1

**DOI:** 10.21956/wellcomeopenres.23364.r85495

**Published:** 2024-06-10

**Authors:** Ruiqi Li

**Affiliations:** 1 University of Colorado Boulder, Boulder, Colorado, USA

Jentoft et al. presented a high-quality genome of the Atlantic cod. I only have a few minor comments.

 1. Introduction: The introduction reads more like results. While it introduces findings from previous versions of the genome, it should also discuss their limitations and explain how the new version improves upon them.

 2. “Here, we showed that Atlantic cod has lost major histocompatibility complex (MHC) II genes,”: Clarify whether this finding is original to this study or based on previous research.

 3. Biological/Ecological Background: Expand on the biological and ecological significance of the species in the introduction.

 4. Mitochondrial Genome: Please double check if the mitochondrial genome was assembled and whether MitoHiFi was used.

 5. Specimen Photo: Include a photo of the specimen used for sequencing

Are sufficient details of methods and materials provided to allow replication by others?

Yes

Is the rationale for creating the dataset(s) clearly described?

Partly

Are the datasets clearly presented in a useable and accessible format?

Yes

Are the protocols appropriate and is the work technically sound?

Yes

Reviewer Expertise:

Genomics

I confirm that I have read this submission and believe that I have an appropriate level of expertise to confirm that it is of an acceptable scientific standard, however I have significant reservations, as outlined above.

# Response

**Published:** 2025-03-02

**Authors:** Tree of Life Team Sanger

**Affiliations:** Wellcome Trust Sanger Institute, UK

Thank you for reviewing this data note. We have responded to your comments below. 

 1. Introduction: The introduction reads more like results. While it introduces findings from previous versions of the genome, it should also discuss their limitations and explain how the new version improves upon them.

***Response: **Thanks for the comment. We have now expanded and rewritten parts of the Introduction/Background, to clarify that we are describing previous results and studies conducted using earlier version of the genome assemblies of Atlantic cod.*

 2. “Here, we showed that Atlantic cod has lost major histocompatibility complex (MHC) II genes,”: Clarify whether this finding is original to this study or based on previous research.

***Response: **As described above, we have now clarified this part of the Introduction, and we have changed the wording here to reflect that we indeed talk about the 2011 study.*

 3. Biological/Ecological Background: Expand on the biological and ecological significance of the species in the introduction.

***Response: **We agree, and we have now expanded this section.*

 4. Mitochondrial Genome: Please double check if the mitochondrial genome was assembled and whether MitoHiFi was used.

***Response: **The mitochondrial genome was not assembled in this particular project/study. MitoHiFi could not be used since this is based on the older Continous Long Read sequencing technology, and not the newer PacBio HiFi/CCS (Circular Consensus Sequence). We have omitted MitoHiFi from the software table.*

 5. Specimen Photo: Include a photo of the specimen used for sequencing.

***Response**: We do not have a photo of the particular specimen used for sequencing suitable in this context. What we have is a poor quality one of a partly dissected individual. We instead use a photo of a representative individual of the same species.*
